# Supplementary material for: Social presence effect in language comprehension: evidence from event-related potential (ERP) research
Source: PeerJ. 2025 Jan 13;13:e18798. doi: 10.7717/peerj.18798 (PMC11737328; doi:10.7717/peerj.18798)
Supplement: Supplemental Information 1 [file peerj-13-18798-s001.docx]

This is the experimental material for this study, and the experimental procedure is presented in Chinese. The first part is the original text of the material and the second part is the corresponding English translation.

| 1 | 何威把金色的子弹装进榨汁机里 |
| --- | --- |
| 2 | 郭颖把新买的杂志放在衣架上 |
| 3 | 张俊打算晚饭之后去散步 |
| 4 | 黄琪把脱下的靴子搁在果盘上 |
| 5 | 徐磊把鲜红的窗花贴在嘴巴上 |
| 6 | 张媛准备写完报告去食堂 |
| 7 | 侯成把崭新的对联贴在大门上 |
| 8 | 贾云把喜欢的浴球扔进垃圾桶里 |
| 9 | 孙梅打算下班之后去看剧 |
| 10 | 钟晖把好用的农药喷洒在庄稼上 |
| 11 | 沈丹把心爱的发卡别到头发上 |
| 12 | 左思把要用的印章按到印泥里 |
| 13 | 江楠把幼小的树苗栽进土坑里 |
| 14 | 叶辰计划上班之前去洗漱 |
| 15 | 郑旭把喜欢的茶叶倒进告示栏里 |
| 16 | 李倩把洗完的衣服晾在下水道 |
| 17 | 陈华准备太阳落山去海滩 |
| 18 | 魏乾把崭新的课本套上被罩 |
| 19 | 马梅把喷完的香水放在钥匙扣上 |
| 20 | 周磊决定这个周末去泰山 |
| 21 | 吕田把要用的充电器插在板凳上 |
| 22 | 董榕把剩下的粉笔放在脖子下 |
| 23 | 谭超把心爱的项链戴在脖子上 |
| 24 | 王刚把许愿的蜡烛插在插座上 |
| 25 | 沙锐把可爱的袜子穿在脚上 |
| 26 | 卢雷把抓到的罪犯关进监狱里 |
| 27 | 顾佳把封好的信封投进邮筒里 |
| 28 | 韩松打算中午过后去招聘会 |
| 29 | 刘洋计划过年放假去采摘 |
| 30 | 范淑把高耸的灯塔建在海岸边 |
| 31 | 蔡敏把剥好的大蒜放进锁里 |
| 32 | 韶涵把配套的椅子放在桌子旁 |
| 33 | 方欢把融化的黄油涂在面包上 |
| 34 | 白宁把破旧的废电池送到回收站 |
| 35 | 杜娇把配好的钥匙穿在钥匙扣上 |
| 36 | 宋璇把清香的洗衣液倒入收纳袋里 |
| 37 | 郝强把珍贵的硬币放入存钱罐里 |
| 38 | 毛微把喜欢的壁纸贴在墙面上 |
| 39 | 姚娜把黑色的墨水灌进钢笔里 |
| 40 | 孟然把开完的轿车停到车库里 |
| 41 | 刘芸计划郊游时去酒庄 |
| 42 | 孙甜决定写完作业去品酒 |
| 43 | 高静把强效的驱蚊贴贴在冰箱上 |
| 44 | 胡雯把捡来的鹅卵石放进录音机里 |
| 45 | 李玲打算有时间去喝酒 |
| 46 | 崔清把冰凉的面膜涂在脸颊上 |
| 47 | 秦涛把鲜活的金鱼放进鱼缸中 |
| 48 | 苏爽把新发的广告贴在茶壶里 |
| 49 | 彭美把干净的浴花放进土坑里 |
| 50 | 雷娟把打开的红酒倒入高脚杯中 |
| 51 | 王成计划今天晚上吃火锅 |
| 52 | 蒋飞把绵软的坐垫放在板凳上 |
| 53 | 陈潇把新买的消炎药放在土坑里 |
| 54 | 许文把干净的毛巾搭在耳朵上 |
| 55 | 常羽把拆开的暖贴贴在衣服上 |
| 56 | 邹洁把回收的铁块投进熔炉里 |
| 57 | 冯慧把晾晒的被子装进花瓶里 |
| 58 | 何华把漂亮的鲜花插在书包里 |
| 59 | 潘玉把收藏的小提琴放回垃圾箱里 |
| 60 | 梁靖把新买的鼠标放在石臼旁 |
| 61 | 贺菁把护眼的台灯摆在书桌上 |
| 62 | 陆萍把灌好的热水袋放进被窝里 |
| 63 | 张华打算今天下午去买书 |
| 64 | 邱瑜把新买的戒指带在手指上 |
| 65 | 尹香把强效的发蜡抹在头发上 |
| 66 | 夏雪把购买的冻鱼放进冰箱里 |
| 67 | 于莉把保暖的围巾系在黑板上 |
| 68 | 石磊把黢黑的煤炭装到炉子里 |
| 69 | 孙晓把削好的铅笔放进饭盒里 |
| 70 | 江杰打算午休期间去画画 |
| 71 | 曾佳把晒干的麦子倒入熔炉里 |
| 72 | 张华准备明天上午去鞋店 |
| 73 | 谢宇把剥好的橘子放进琴盒里 |
| 74 | 任健把掉落的镜片安回镜框上 |
| 75 | 袁希把地上的垃圾捡到琴盒里 |
| 76 | 刘斌把吃完的剩饭放进文具袋里 |
| 77 | 严笑把沉重的牌匾挂在门楣上 |
| 78 | 曹雪把老旧的磁带放进花盆里 |
| 79 | 廖怡把烧好的蹄铁镶在马蹄上 |
| 80 | 王鹏准备明天上午去买药 |
| 81 | 钱聪把干净的纱布包在伤口处 |
| 82 | 万鹏把重要的日期写在日历上 |
| 83 | 程娴把正确的钥匙插到澡篮里 |
| 84 | 叶俊把干净的铁锅放在皇冠上 |
| 85 | 金叹把心爱的耳环带在耳朵上 |
| 86 | 戴哲把修好的路牌立在道路旁 |
| 87 | 梁萌打算上午有空去赌博 |
| 88 | 尹兵把刚办的银行卡放进卡包里 |
| 89 | 陈萍决定看完电视剧去买菜 |
| 90 | 吴芸把心爱的摩托车停在手枪里 |
| 91 | 牛尧把合适的扣子钉在衣服上 |
| 92 | 姜迪把漂亮的宝石镶在皇冠上 |
| 93 | 武珂把刚买的种子种进花盆里 |
| 94 | 邓睿把烧开的热水倒进药箱里 |
| 95 | 梁颖准备这个周末去买药 |
| 96 | 赵军把新换的手机贴上被罩 |
| 97 | 彭力把拼好的模型摆入火锅里 |
| 98 | 宋悦计划今天下午去练琴 |
| 99 | 张伟把得到的奖状贴在鞋架上 |
| 100 | 孔琦把用完的卫生纸扔进纸篓里 |
| 101 | 楚秀把滋润的口红涂在嘴唇上 |
| 102 | 李明把刚买的葡萄放进鞋架上 |
| 103 | 田珊把要用的优盘插在电脑上 |
| 104 | 薛辉把喝完的饮料瓶扔进保温箱 |
| 105 | 韩松把网购的脚垫放在蛋糕外 |
| 106 | 熊茜把成箱的汽油倒入油箱里 |
| 107 | 罗越把美味的肥牛卷放进展柜里 |
| 108 | 霍曼把重要的棋子放在棋盘上 |
| 109 | 林浩把刷好的盘子放进手枪里 |
| 110 | 乔英把打开的饮料倒进杯子里 |
| 111 | 曲霞把新买的口红涂在毛巾架上 |
| 112 | 徐明准备下午有空去喝茶 |
| 113 | 栗冉把想听的唱片放到留声机里 |
| 114 | 赵明决定周六晚上去唱歌 |
| 115 | 郭欣把网购的脚垫放在窗户外 |
| 116 | 汪晨把新鲜的鱼饵挂在鱼钩上 |
| 117 | 唐利把晾干的被子套上钢化膜 |
| 118 | 杨悦把抢购的雪糕放进车库里 |
| 119 | 王茜决定放学之后去学习 |
| 120 | 张伟准备中午之后去陵园 |
| 121 | 朱阳把要用的课本装进橱柜里 |
| 122 | 田乐打算周五晚上去按摩店 |
| 123 | 丁巧把好用的牙膏挤在牙刷上 |
| 124 | 周璐把办好的护照套上发带 |
| 125 | 汪萍把拍好的照片放进冰箱 |
| 126 | 彭力把拼好的模型摆入展柜里 |
| 127 | 乔英把打开的饮料倒进油箱里 |
| 128 | 顾佳把封好的保鲜袋投进邮筒里 |
| 129 | 毛微把喜欢的壁纸贴在衣服上 |
| 130 | 苏燕计划午饭之后去听戏 |
| 131 | 姚娜把黑色的墨水灌进监狱里 |
| 132 | 孟然把开完的轿车停到车库里 |
| 133 | 汪晨把新鲜的鱼饵挂在耳朵上 |
| 134 | 丁巧把好用的牙膏挤在头发上 |
| 135 | 栗冉把想听的唱片放到印泥里 |
| 136 | 赵军打算做完工作去祷告 |
| 137 | 万鹏把重要的日期写在门楣上 |
| 138 | 叶俊把干净的铁锅放在灶台上 |
| 139 | 姜迪把漂亮的宝石镶在庄稼上 |
| 140 | 袁希把地上的垃圾捡到垃圾箱里 |
| 141 | 方欢把融化的黄油涂在日历上 |
| 142 | 陈军计划腾出时间去理发 |
| 143 | 胡雯把捡来的鹅卵石放进花盆里 |
| 144 | 白宁把破旧的废电池送到海岸边 |
| 145 | 朱阳把要用的课本装进书包里 |
| 146 | 于莉把保暖的围巾系在脖子上 |
| 147 | 高静把强效的驱蚊贴贴在蚊帐上 |
| 148 | 李霞决定吃完晚饭去邮局 |
| 149 | 曹雪把老旧的磁带放进录音机里 |
| 150 | 郭颖把新买的杂志放在书架上 |
| 151 | 侯成把崭新的对联贴在脖子上 |
| 152 | 韶涵把配套的椅子放在墙面上 |
| 153 | 陈丽打算周五晚上去健身 |
| 154 | 林爽准备下班之后去取钱 |
| 155 | 雷娟把打开的红酒倒入存钱罐里 |
| 156 | 程娴把正确的钥匙插到锁里 |
| 157 | 秦涛把鲜活的金鱼放进鱼缸中 |
| 158 | 刘宇计划完成培训去考古 |
| 159 | 蒋飞把绵软的坐垫放在插座上 |
| 160 | 孙晓把削好的铅笔放进文具袋 |
| 161 | 夏雪把购买的冻鱼放进相册里 |
| 162 | 赵军决定工作之后去球场 |
| 163 | 牛尧把合适的扣子钉在头发上 |
| 164 | 郑旭把喜欢的茶叶倒进茶壶里 |
| 165 | 邱瑜把新买的戒指带在马蹄上 |
| 166 | 魏乾把崭新的课本套上书皮 |
| 167 | 胡珊决定明天早上去补课 |
| 168 | 张超准备这个周末去游泳 |
| 169 | 谭超把心爱的项链戴在大门上 |
| 170 | 王刚把许愿的蜡烛插在蛋糕上 |
| 171 | 李倩把洗完的衣服晾在衣架上 |
| 172 | 霍曼把重要的棋子放在脚上 |
| 173 | 唐利把晾干的被子套上被罩 |
| 174 | 陈婷准备做完家务去打牌 |
| 175 | 邓宇计划下周休息去步行街 |
| 176 | 李明把刚买的葡萄放进果盘里 |
| 177 | 杜娇把配好的钥匙穿在电脑上 |
| 178 | 沙锐把可爱的袜子穿在棋盘上 |
| 179 | 金叹把心爱的耳环带在鱼钩上 |
| 180 | 曲霞把新买的口红涂在嘴巴上 |
| 181 | 黄静计划拿到工资去文眉 |
| 182 | 郭欣把网购的脚垫放在浴室外 |
| 183 | 何华把漂亮的鲜花插在花瓶里 |
| 184 | 武珂把刚买的种子种进卡包里 |
| 185 | 陈潇把新买的消炎药放在药箱里 |
| 186 | 吴芸把心爱的摩托车停在车库里 |
| 187 | 熊茜把成箱的汽油倒入垃圾桶里 |
| 188 | 潘玉把收藏的小提琴放回琴盒里 |
| 189 | 曾佳把晒干的麦子倒入粮仓里 |
| 190 | 刘明计划运动之后去洗澡 |
| 191 | 张瑾决定周日上午去观星 |
| 192 | 谢宇把剥好的橘子放进榨汁机里 |
| 193 | 尹兵把刚办的银行卡放进花盆里 |
| 194 | 汤雯决定午休之后去取件 |
| 195 | 马梅把喷完的香水放在梳妆台上 |
| 196 | 田珊把要用的优盘插在钥匙扣上 |
| 197 | 张铭计划这个周末去钓鱼 |
| 198 | 刘晶准备今天傍晚去温泉 |
| 199 | 任健把掉落的镜片安回海岸边 |
| 200 | 张伟把得到的奖状贴在墙上 |
| 201 | 邓睿把烧开的热水倒进暖水瓶 |
| 202 | 袁静计划工作结束去烧烤店 |
| 203 | 戴哲把修好的路牌立在道路旁 |
| 204 | 赵军把新换的手机贴上钢化膜 |
| 205 | 彭美把干净的浴花放进澡篮里 |
| 206 | 孔琦把用完的卫生纸扔进留声机里 |
| 207 | 严笑把沉重的牌匾挂在日历上 |
| 208 | 许文把干净的毛巾搭在毛巾架上 |
| 209 | 刘斌把吃完的剩饭放进冰箱里 |
| 210 | 钟晖把好用的农药喷洒在皇冠上 |
| 211 | 吕田把要用的充电器插在插座上 |
| 212 | 贾云把喜欢的浴球扔进浴缸里 |
| 213 | 周璐把办好的护照套上护照夹 |
| 214 | 徐磊把鲜红的窗花贴在窗户上 |
| 215 | 罗越把美味的肥牛卷放进火锅里 |
| 216 | 邹洁把回收的铁块投进熔炉里 |
| 217 | 郝强把珍贵的硬币放入高脚杯中 |
| 218 | 张妍决定下班之后去美甲店 |
| 219 | 方雪决定周末晚上去跳舞 |
| 220 | 宋璇把清香的洗衣液倒入洗衣机里 |
| 221 | 左思把要用的印章按到垃圾桶里 |
| 222 | 张鹏打算下课之后去网吧 |
| 223 | 卢雷把抓到的罪犯关进钢笔里 |
| 224 | 冯东决定明天早上去植物园 |
| 225 | 李梅打算下班之后去购物 |
| 226 | 范淑把高耸的灯塔建在镜框上 |
| 227 | 陆萍把灌好的热水袋放进被窝里 |
| 228 | 薛辉把喝完的饮料瓶扔进垃圾桶 |
| 229 | 楚秀把滋润的口红涂在衣服上 |
| 230 | 汪萍把拍好的照片放进相册里 |
| 231 | 冯慧把晾晒的被子装进收纳袋里 |
| 232 | 廖怡把烧好的蹄铁镶在手指上 |
| 233 | 尹香把强效的发蜡抹在嘴唇上 |
| 234 | 韩松把网购的脚垫放在浴室外 |
| 235 | 江楠把幼小的树苗栽进被窝里 |
| 236 | 常羽把拆开的暖贴贴在头发上 |
| 237 | 董榕把剩下的粉笔放在黑板下 |
| 238 | 苏爽把新发的广告贴在告示栏里 |
| 239 | 崔清把冰凉的面膜涂在脸颊上 |
| 240 | 贺菁把护眼的台灯摆在面包上 |
| 241 | 钱聪把干净的纱布包在办事处 |
| 242 | 王娟准备有空时去拜佛 |
| 243 | 杨悦把抢购的雪糕放进冰箱里 |
| 244 | 沈丹把心爱的发卡别到牙刷上 |
| 245 | 何威把金色的子弹装进手枪里 |
| 246 | 石磊把黢黑的煤炭装到炉子里 |
| 247 | 蔡敏把剥好的大蒜放进石臼里 |
| 248 | 林浩把刷好的盘子放进橱柜里 |
| 249 | 梁靖把新买的鼠标放在电脑旁 |
| 250 | 黄琪把脱下的靴子搁在鞋架上 |

| 1 | He Wei loaded the golden bullet into the juicer |
| --- | --- |
| 2 | Guo Ying puts her newly bought magazines on a hanger |
| 3 | Zhang Jun plans to go for a walk after dinner |
| 4 | Huang Qi rested his boots on the fruit plate |
| 5 | Xu Lei put the bright red window grille on his mouth |
| 6 | Zhang Yuan was about to finish writing the report and go to the cafeteria |
| 7 | Hou Cheng pasted the brand new couplet on the door |
| 8 | Jia Yun threw his favorite bath ball into the trash |
| 9 | Sun Mei plans to go to the drama after work |
| 10 | Zhong Hui sprayed the crops with good pesticides |
| 11 | Shen Dan pinned his beloved hairpin to his hair |
| 12 | Zuo Si pressed the seal to be used into the ink |
| 13 | Jiang Nan planted the young saplings in the soil pit |
| 14 | Ye Chen planned to wash up before going to work |
| 15 | Zheng Xu poured his favorite tea leaves into the notice board |
| 16 | Li Qian hung the washed clothes in the sewer |
| 17 | Chen Hua prepares for the sun to go down to the beach |
| 18 | Wei Qian put the brand-new textbook on the cover |
| 19 | Mame puts the finished perfume on the keychain |
| 20 | Zhou Lei decided to go to Mount |
| 21 | Lu Tian plugged the charger he wanted to use on the bench |
| 22 | Dong Rong put the remaining chalk under his neck |
| 23 | Tan Chao wore his beloved necklace around his neck |
| 24 | Wang Gang inserted the wishing candle into the socket |
| 25 | Sha Rui wears cute socks on her feet |
| 26 | Luray put the criminals he caught in prison |
| 27 | Gu Jia threw the sealed envelope into the mailbox |
| 28 | Han Song planned to go to the job fair after noon |
| 29 | Liu Yang plans to go picking during the Chinese New Year holiday |
| 30 | Fan Shu built a towering lighthouse on the shore |
| 31 | Cai Min put the peeled garlic into the lock |
| 32 | Shao Han placed the matching chair next to the table |
| 33 | Fang Huan spread the melted butter on the bread |
| 34 | Bai Ning sends the worn-out waste batteries to the recycling station |
| 35 | Du Jiao put the matching key on the keychain |
| 36 | Song Xuan poured the fragrant laundry detergent into the storage bag |
| 37 | Hao Qiang put the precious coins in the piggy bank |
| 38 | Mao Wei pasted her favorite wallpaper on the wall |
| 39 | Yao Na poured black ink into the pen |
| 40 | Meng Ran parked the finished car in the garage |
| 41 | Liu Yun plans to go to the winery during an outing |
| 42 | Sun Tian decided to finish his homework and go for a wine tasting |
| 43 | Gao Jing put a powerful mosquito repellent patch on the refrigerator |
| 44 | Hu Wen put the pebbles she had picked up into the tape recorder |
| 45 | Li Ling plans to have time to drink |
| 46 | Cui Qing applied a cold mask to his cheeks |
| 47 | Qin Tao put the live goldfish into the fish tank |
| 48 | Su Shuang posted the new advertisement in the teapot |
| 49 | Peng Mei put the clean bath flowers into the dirt pit |
| 50 | Lei Juan poured the opened red wine into the goblet |
| 51 | Wang Cheng plans to eat hot pot tonight |
| 52 | Jiang Fei put the soft cushion on the bench |
| 53 | Chen Xiao put the newly bought anti-inflammatory medicine in the earthen pit |
| 54 | Xu Wen put a clean towel on his ear |
| 55 | Chang Yu put the dismantled warm patch on his clothes |
| 56 | Zou Jie threw the recovered iron into the furnace |
| 57 | Feng Hui put the drying quilt into a vase |
| 58 | He Hua put beautiful flowers in his school bag |
| 59 | Pan Yu put the violin back in the trash |
| 60 | Liang Jing put the newly bought mouse next to the stone mortar |
| 61 | He Jing placed the eye-protecting lamp on the desk |
| 62 | Lu Ping put the filled hot water bottle into the quilt |
| 63 | Zhang Hua plans to go buy books this afternoon |
| 64 | Qiu Yu put the newly bought ring on his finger |
| 65 | Yin Xiang applied a powerful wax to her hair |
| 66 | Xia Xue put the frozen fish she bought in the refrigerator |
| 67 | Yu Li tied her warm scarf to the blackboard |
| 68 | Shi Lei loaded the black coal into the furnace |
| 69 | Sun Xiao put the sharpened pencil into the lunch box |
| 70 | Jiang Jie plans to paint during his lunch break |
| 71 | Zeng Jia poured the dried wheat into the furnace |
| 72 | Zhang Hua is going to the shoe store tomorrow morning |
| 73 | Xie Yu put the peeled orange into the piano case |
| 74 | Ren Jian put the dropped lens back on the frame |
| 75 | Yuan Xi picked up the garbage on the ground and put it in the piano case |
| 76 | Liu Bin put the leftovers into the stationery bag |
| 77 | Yan Xiao hung the heavy plaque on the lintel |
| 78 | Cao Xue put the old tape into the flower pot |
| 79 | Liao Yi set the burnt farrier on the horse's hooves |
| 80 | Wang Peng is going to buy medicine tomorrow morning |
| 81 | Qian Cong wrapped clean gauze around the wound |
| 82 | Wan Peng writes important dates on the calendar |
| 83 | Cheng Xian inserted the correct key into the bath basket |
| 84 | Ye Jun put the clean iron pot on the crown |
| 85 | Jin Si put his beloved earrings on his ears |
| 86 | Dai Zhe put the repaired street sign on the side of the road |
| 87 | Liang Meng plans to gamble in the morning |
| 88 | Yin Bing put the bank card he had just opened into the card bag |
| 89 | Chen Ping decided to go grocery shopping after watching the TV series |
| 90 | Wu Yun parked her beloved motorcycle in a pistol |
| 91 | Niu Yao nailed the appropriate buttons to his clothes |
| 92 | Jiang Di set the beautiful gem in the crown |
| 93 | Wu Ke planted the seeds he had just bought into a pot |
| 94 | Deng Rui poured the boiling hot water into the medicine box |
| 95 | Liang Ying is going to buy medicine this weekend |
| 96 | Zhao Jun put the new mobile phone on the quilt cover |
| 97 | Peng Li put the assembled model into the hot pot |
| 98 | Song Yue plans to practice this afternoon |
| 99 | Zhang Wei pasted the award on the shoe rack |
| 100 | Kong Qi threw the used toilet paper into the paper basket |
| 101 | Chu Xiu applied the moisturizing lipstick to her lips |
| 102 | Li Ming put the grapes he had just bought on the shoe rack |
| 103 | Tian Shan plugged the USB drive she wanted to use into the computer |
| 104 | Xue Hui threw the finished drink bottle into the incubator |
| 105 | Han Song put the mat he bought online on the outside of the cake |
| 106 | Xiong Qian poured boxes of gasoline into the tank |
| 107 | Luo Yue put the delicious fat beef rolls in the cabinet |
| 108 | Homan placed important pieces on the board |
| 109 | Lin Hao put the brushed plate into the pistol |
| 110 | Qiao Ying poured the opened drink into the cup |
| 111 | Qu Xia put her newly bought lipstick on the towel rack |
| 112 | Xu Ming was going to have time to drink tea in the afternoon |
| 113 | Li Ran put the record he wanted to listen to into the gramophone |
| 114 | Zhao Ming decided to go singing on Saturday night |
| 115 | Guo Xin put the mat she bought online outside the window |
| 116 | Wang Chen hung fresh bait on the hook |
| 117 | Tang Li put the dried quilt on tempered film |
| 118 | Yang Yue put the snapped ice cream into the garage |
| 119 | Wang Qian decided to go to study after school |
| 120 | Zhang Wei was going to the cemetery after noon |
| 121 | Zhu Yang put the textbooks he wanted to use into the cupboard |
| 122 | Tian Le plans to go to the massage parlor on Friday night |
| 123 | Ding Qiao squeezed the useful toothpaste on the toothbrush |
| 124 | Zhou Lu put the completed passport on the headband |
| 125 | Wang Ping put the photos in the refrigerator |
| 126 | Peng Li put the assembled model into the display case |
| 127 | Qiao Ying poured the open drink into the gas tank |
| 128 | Gu Jia threw the sealed fresh-keeping bag into the mailbox |
| 129 | Mao Wei pasted her favorite wallpaper on her clothes |
| 130 | Su Yan planned to go to the theater after lunch |
| 131 | Yao Na poured black ink into the prison |
| 132 | Meng Ran parked the finished car in the garage |
| 133 | Wang Chen hung fresh bait on his ears |
| 134 | Ding Qiao squeezed the good toothpaste into his hair |
| 135 | Li Ran put the record he wanted to listen to into the ink |
| 136 | Zhao Jun planned to finish his work and pray |
| 137 | Wan Peng wrote important dates on the lintel |
| 138 | Ye Jun put the clean iron pot on the stove |
| 139 | Jiang Di set beautiful gems on the crops |
| 140 | Yuan Xi picked up the garbage on the ground and put it in the garbage bin |
| 141 | Fang Huan smeared the melted butter on the calendar |
| 142 | Chen Jun plans to make time for a haircut |
| 143 | Hu Wen put the pebbles she picked up into the flower pot |
| 144 | Bai Ning sent the worn-out waste batteries to the coast |
| 145 | Zhu Yang put the textbooks he wanted to use into his schoolbag |
| 146 | Yu Li tied a warm scarf around her neck |
| 147 | Gao Jing put a powerful mosquito repellent patch on the mosquito net |
| 148 | Li Xia decided to go to the post office after dinner |
| 149 | Cao Xue put the old tape into the tape recorder |
| 150 | Guo Ying puts her newly bought magazines on the shelves |
| 151 | Hou Cheng put the brand new couplet around his neck |
| 152 | Shao Han placed the matching chair on the wall |
| 153 | Chen Li plans to go to the gym on Friday night |
| 154 | Lin Shuang was going to withdraw the money after work |
| 155 | Lei Juan poured the opened red wine into the piggy bank |
| 156 | Cheng Xian inserted the correct key into the lock |
| 157 | Qin Tao put the live goldfish into the fish tank |
| 158 | Liu Yu plans to complete his training to go to archaeology |
| 159 | Jiang Fei put the soft cushion on the socket |
| 160 | Sun Xiao put the sharpened pencil into the stationery bag |
| 161 | Xia Xue put the frozen fish she bought into the album |
| 162 | Zhao Jun decided to go to the stadium after work |
| 163 | Niu Yao nailed the appropriate buttons to his hair |
| 164 | Zheng Xu poured his favorite tea leaves into the teapot |
| 165 | Qiu Yu put the newly bought ring on the horse's hooves |
| 166 | Wei Qian put the brand-new textbook on the book cover |
| 167 | Hu Shan decided to go to make up for the class tomorrow morning |
| 168 | Zhang Chao is ready to go swimming this weekend |
| 169 | Tan Chao wore his beloved necklace on the door |
| 170 | Wang Gang put the wishing candle on the cake |
| 171 | Li Qian hung the washed clothes on the hanger |
| 172 | Hörmann puts important pieces on his feet |
| 173 | Donley put the dried quilt on the cover |
| 174 | Chen Ting was ready to finish her housework and go to play cards |
| 175 | Deng Yu plans to take a break next week to go to the pedestrian street |
| 176 | Li Ming put the grapes he had just bought into the fruit bowl |
| 177 | Du Jiao put the matching key on the computer |
| 178 | Sha Rui wears cute socks on the chessboard |
| 179 | Jin Si wears her beloved earrings on the fishhook |
| 180 | Qu Xia put the newly bought lipstick on her mouth |
| 181 | Huang Jing plans to get her salary and go to Wenmei |
| 182 | Guo Xin put the mat she bought online outside the bathroom |
| 183 | He Hua put beautiful flowers in a vase |
| 184 | Wu Ke planted the seeds he had just bought into the card pack |
| 185 | Chen Xiao put the newly bought anti-inflammatory medicine in the medicine box |
| 186 | Wu Yun parked her beloved motorcycle in the garage |
| 187 | Xiong Qian emptied boxes of gasoline into the trash |
| 188 | Pan Yu put the violin back in the case |
| 189 | Zeng Jia poured the dried wheat into the granary |
| 190 | Liu Ming plans to take a shower after exercising |
| 191 | Zhang Jin decided to go stargazing on Sunday morning |
| 192 | Xie Yu put the peeled oranges into the juicer |
| 193 | Yin Bing put the bank card he had just opened into a flower pot |
| 194 | Tang Wen decided to pick up the parcel after her lunch break |
| 195 | Mame put the finished perfume on the dresser |
| 196 | Tian Shan inserted the USB drive he wanted to use into the keychain |
| 197 | Zhang Ming plans to go fishing this weekend |
| 198 | Liu Jing is going to go to the hot spring this evening |
| 199 | Ren Jian returned the dropped lens to the shore |
| 200 | Zhang Wei posted the award certificate on the wall |
| 201 | Deng Rui poured boiling hot water into the thermos |
| 202 | Yuan Jing plans to go to the barbecue restaurant after work |
| 203 | Dai Zhe put the repaired street sign on the side of the road |
| 204 | Zhao Jun put the new mobile phone with tempered film |
| 205 | Peng Mei put the clean bath flowers in the bath basket |
| 206 | Kong Qi threw the used toilet paper into the gramophone |
| 207 | Yan Xiao hung the heavy plaque on the calendar |
| 208 | Xu Wen put the clean towel on the towel rack |
| 209 | Liu Bin put the leftovers in the refrigerator |
| 210 | Zhong Hui sprayed the good pesticide on the crown |
| 211 | Lu Tian plugged the charger he wanted to use into the socket |
| 212 | Jia Yun threw his favorite bath ball into the bathtub |
| 213 | Zhou Lu put the completed passport on the passport holder |
| 214 | Xu Lei pasted the bright red window grille on the window |
| 215 | Luo Yue put the delicious fat beef rolls into the hot pot |
| 216 | Zou Jie threw the recovered iron into the furnace |
| 217 | Hao Qiang put the precious coin into the goblet |
| 218 | Zhang Yan decided to go to the nail salon after work |
| 219 | Fang Xue decided to go dancing on weekend nights |
| 220 | Song Xuan poured the fragrant laundry detergent into the washing machine |
| 221 | Zuo Si pressed the seal he wanted to use into the trash |
| 222 | Zhang Peng plans to go to an Internet café after class |
| 223 | Luray put the criminals he caught in pens |
| 224 | Feng Dong decided to go to the Botanical Garden tomorrow morning |
| 225 | Li Mei plans to go shopping after work |
| 226 | Fan Shu built the towering lighthouse on the frame |
| 227 | Lu Ping put the filled hot water bottle into the quilt |
| 228 | Xue Hui threw the finished drink bottle into the trash |
| 229 | Chu Xiu put the moisturizing lipstick on his clothes |
| 230 | Wang Ping put the photos she had taken into the album |
| 231 | Feng Hui put the drying quilt into a storage bag |
| 232 | Liao Yi put the burnt farrier on his finger |
| 233 | Yin Xiang applied the powerful hair wax to her lips |
| 234 | Han Song put the mat he bought online outside the bathroom |
| 235 | Jiang Nan planted the young saplings in the quilt |
| 236 | Chang Yu put the disassembled warm patch on his hair |
| 237 | Dong Rong put the rest of the chalk under the blackboard |
| 238 | Su Shuang posted the new advertisement on the notice board |
| 239 | Cui Qing applied a cold mask to his cheeks |
| 240 | He Jing placed the eye-protecting lamp on the bread |
| 241 | Qian Cong wrapped clean gauze in the office |
| 242 | Wang Juan is ready to worship the Buddha when she has time |
| 243 | Yang Yue put the snapped ice cream into the refrigerator |
| 244 | Shen Dan pinned his beloved hairpin to the toothbrush |
| 245 | He Wei loaded the golden bullet into the pistol |
| 246 | Shi Lei loaded the black coal into the furnace |
| 247 | Cai Min put the peeled garlic into a stone mortar |
| 248 | Lin Hao put the brushed plate into the cupboard |
| 249 | Liang Jing put the newly bought mouse next to the computer |
| 250 | Huang Qi rested the boots he had taken off on the shoe rack |

This is the questionnaire mentioned in the paper (viewed by the subjects together with others present), presented in Chinese during the experiment. The first part is the original questionnaire and the second part is the corresponding English translation.

**测试：** 下面出现的词哪些是你刚刚记忆过的？

木讷

悲恸

愕然

展柜

疲惫

油箱

羞涩

监狱

惊恐

书包

悲壮

祷告

疲倦

欢愉

果盘

愤慨

鱼钩

疲乏

皇冠

羞愧

心浮

惊骇

可耻

心寒

熔炉

跳舞

哀怨

困惑

腼腆

石臼

黢黑

晾晒

强效

**Test：** Which of the following words have you just memorized?

dull

grief

stunned

showcase

exhaustion

fuel tank

shy

jail

afraid

schoolbag

tragic

pray

tired

joy

fruit

indignation

fishhook

tired

crown

mortified

the heart floats

horror

shameful

chilling

furnace

dance

plaintive

confused

shy

stone mortar

dark

drying

potency
